# Causes and costs of global COVID-19 vaccine inequity

**DOI:** 10.1007/s00281-023-00998-0

**Published:** 2023-10-23

**Authors:** Maddalena Ferranna

**Affiliations:** Department of Pharmaceutical and Health Economics, Alfred E. Mann School of Pharmacy and Pharmaceutical Sciences, University of Southern California, Los Angeles, CA USA

**Keywords:** Vaccination, Equity, COVID-19, Nationalism, Vaccine hesitancy, Socioeconomic costs

## Abstract

Despite the rapid development of safe and effective COVID-19 vaccines and the widely recognized health and economic benefits of vaccination, there exist stark differences in vaccination rates across country income groups. While more than 70% of the population is fully vaccinated in high-income countries, vaccination rates in low-income countries are only around 30%. The paper reviews the factors behind global COVID-19 vaccine inequity and the health, social, and economic costs triggered by this inequity. The main contributors to vaccine inequity include vaccine nationalism, intellectual property rights, constraints in manufacturing capacity, poor resilience of healthcare systems, and vaccine hesitancy. Vaccine inequity has high costs, including preventable deaths and cases of illnesses in low-income countries, slow economic recovery, and large learning losses among children. Increasing vaccination rates in low-income countries is in the self-interest of higher-income countries as it may prevent the emergence of new variants and continuous disruptions to global supply chains.

## Introduction

The COVID-19 pandemic triggered staggering health, social, and economic costs. As of July 2023, there have been more than 6.9 million confirmed COVID-19 deaths worldwide [1], although estimates of excess mortality suggest that the total number of deaths caused by the pandemic (i.e., including also deaths from other causes) is two to three times larger than the official count [2, 3]. The discrepancy is due to likely under-reporting of COVID-19 deaths [4], but also to the broad health impacts of the pandemic, which encompassed the overtaxing of healthcare systems [5, 6], the disruption of routine immunization and medical testing [7], delays in treatments [8], and reduced willingness to seek care out of fear of infection or pandemic-related negative income shocks (e.g., unemployment and health insurance loss) [9]. The COVID-19 pandemic is also responsible for the largest global economic crisis seen in a century [10]. The pandemic ushered in school and workplace closures to control the spread of infections, driving massive unemployment, permanent business closures, disruptions in global supply chains, and increasing learning gaps between rich and poor children [11–15]. The International Monetary Fund (IMF) estimates that global gross domestic product (GDP) dropped by 3.1% during 2020 [16]. Even though the global economy recovered during 2021 (with global GDP growing by 6%) thanks to access to COVID-19 vaccines and the relaxation of social and economic restrictions, the negative socioeconomic effects of the pandemic are lingering and contribute to the economic slowdowns experienced in 2022 and projected for 2023 [17].

The rapid development of effective and safe COVID-19 vaccines played a key role in containing the health and socioeconomic costs of the pandemic [18]. Vaccination allowed countries to control the spread of infections and to reduce hospitalizations, mortality, and morbidity. It has also allowed countries to reopen their economies and to ease mobility restrictions. While the health and socioeconomic benefits of COVID-19 vaccines are unquestionable [19], COVID-19 vaccination rollout has proceeded at heterogeneous paces throughout the world. As of July 2023, approximately 65% of the global population has completed the initial COVID-19 vaccination protocol (i.e., two shots for most vaccines). However, while in high-income countries, more than 70% of their population have completed the initial protocol; among low-income and lower-middle-income countries, vaccination rates are falling behind. In particular, only 2% of total COVID-19 vaccine doses (including boosters) have been administered in low-income countries [20].

Low-income and lower-middle-income countries have relied mostly on vaccine donations from developed countries and on support from COVAX (COVID-19 Vaccines Global Access), a global vaccine sharing scheme led by the Coalition for Epidemic Preparedness Innovations (CEPI), Gavi the Vaccine Alliance, and the World Health Organization (WHO) [21]. As of July 2023, COVAX has delivered almost 1.9 billion doses of COVID-19 vaccines to 146 countries, mostly low- and lower-middle-income countries [22]. Nonetheless, vaccination rates have proceeded at very different speeds across countries’ income groups. Several factors have undermined the effective delivery of vaccines through COVAX, including vaccine hoarding, manufacturing constraints, export bans, and logistical issues [23]. Spreading vaccine hesitancy due to misinformation or mistrust contributes to low vaccination uptakes [24].

The persistent global inequity in vaccination rates has daunting health, social, and economic consequences [25]. Low vaccination rates have caused preventable deaths and illnesses, slowed down the economic recovery process, and exacerbated socioeconomic inequities both within and between countries, among other factors. Low vaccination rates make countries vulnerable to the risk of new infection waves in the future, with the resulting stream of health, social, and economic burdens.

The objective of the paper is to examine the health, social, and economic costs caused by COVID-19 vaccine inequity and to draw lessons for pandemic preparedness. Using results from the literature and publicly available datasets, the paper will first describe patterns of COVID-19 vaccine allocation across countries and over time and characterize the degree of inequity in the distribution of vaccines. The paper will then discuss the factors that contributed to inequity in the global allocation of COVID-19 vaccines and examine the health, social, and economic costs caused by this inequity.

## Inequity in the global allocation of COVID-19 vaccines

**Fig 1:**
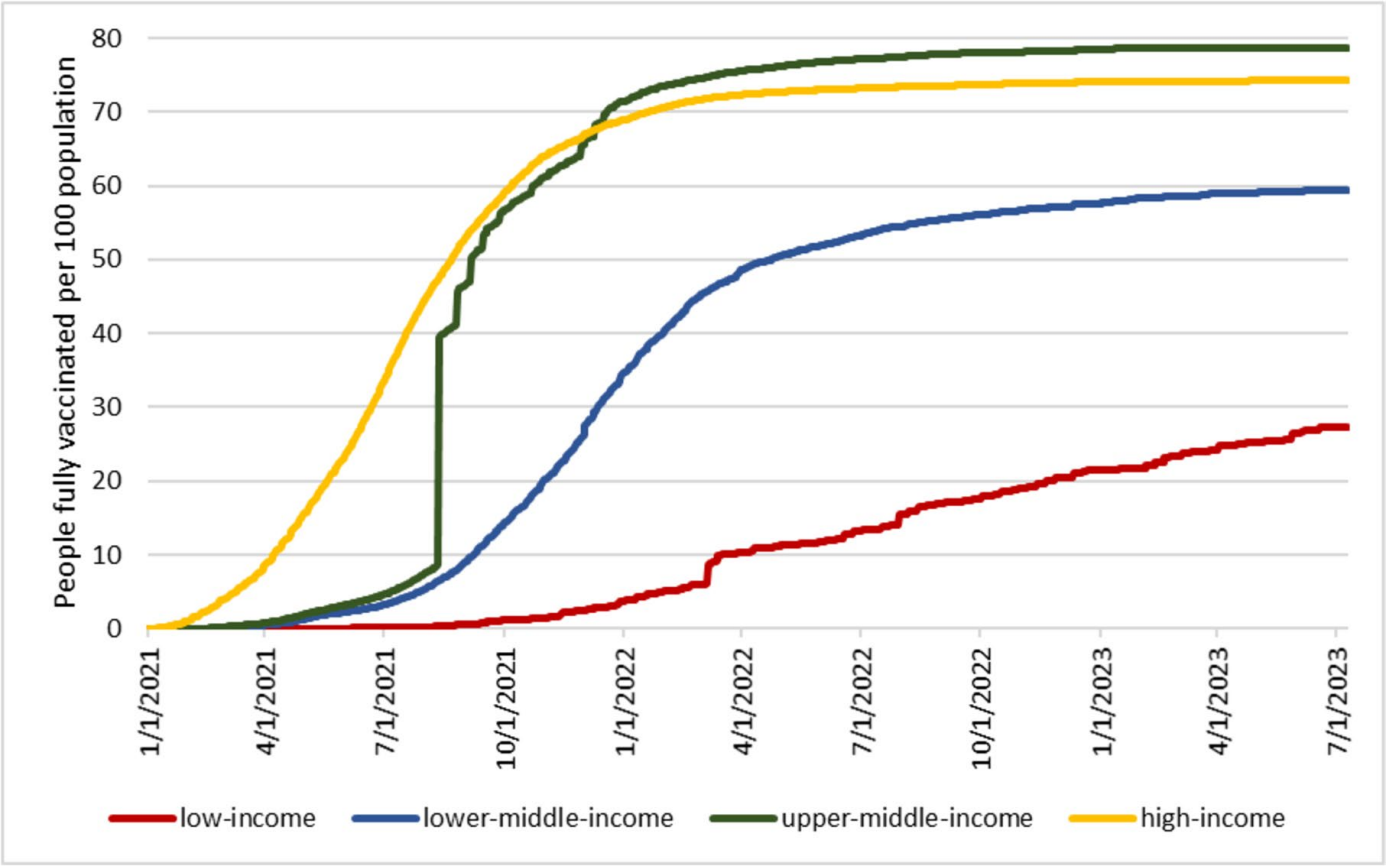
Percentage of fully vaccinated individuals by income group. Source: Our World in Data [20]. The classification of countries by income is based on the 2021 World Bank definition of income groups

COVID-19 vaccination coverage varies starkly across countries. Figure 1 depicts the percentage of people fully vaccinated against COVID-19 by countries’ income group: For each income group and date, I divided the cumulative number of fully vaccinated individuals by the total population of that income group. Individuals are considered fully vaccinated if they completed the initial vaccination protocol (three doses in three-dose vaccines, two doses in two-dose vaccines, and one dose in one-dose vaccines). As of July 2023, 75% of people in high-income countries and 79% of people in upper-middle-income countries have been fully vaccinated. In lower-middle-income countries, the percentage of fully vaccinated individuals is around 59%. In contrast, in low-income countries, only 27% of the population has been fully vaccinated. The time profile of vaccinations also differs markedly across countries, with higher-income countries starting the vaccination process earlier than lower-income countries. Note that Figure 1 is based on countries’ official reports of vaccinations. The discontinuities in the time patterns of vaccination reflect discontinuities in the timing of reports.

In 2021, the World Health Organization recommended that countries should have at least 70% of their population fully vaccinated by the end of June 2022 [26]. This target was set to guarantee global COVID-19 protection as 70% was considered a good estimate of the herd immunity threshold (i.e., the minimum proportion of COVID-19 vaccinations that would allow interruption of the chain of transmissions). As Figure 1 shows, only high-income and upper-middle-income countries achieved that goal. Even though COVID-19 herd immunity is now considered an elusive goal due to the continuous emergence of new variants that escape infection-acquired and vaccination-acquired immunity [27], the comparison with the 70% vaccination coverage level is still informative about the large inequality in vaccination coverage across countries.

**Fig 2:**
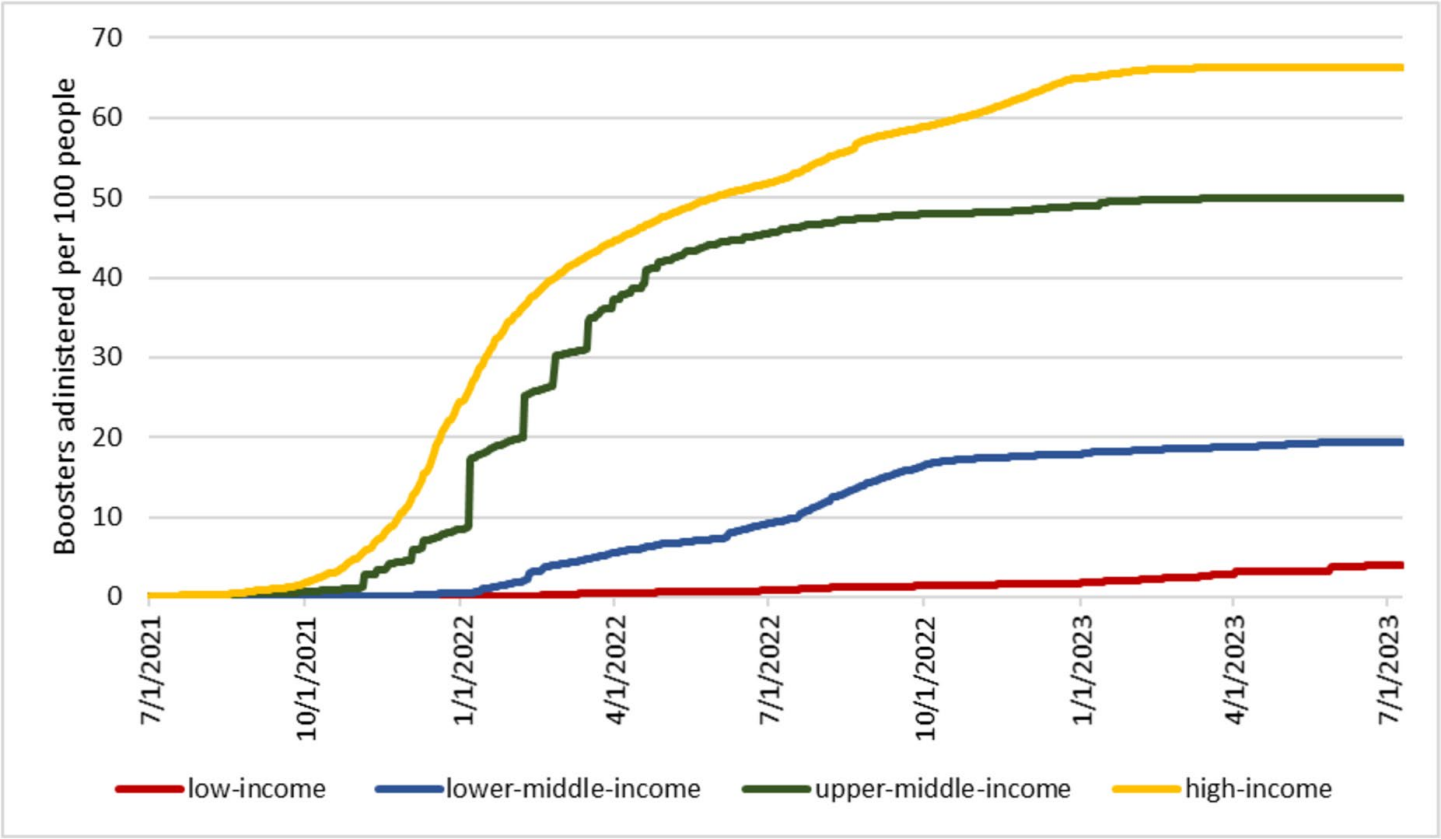
COVID-19 vaccine boosters administered per 100 people by country income group. Source: Our World in Data [20]

Figure 1 looks at full vaccinations. Instead, Figure 2 depicts the number of COVID-19 vaccine boosters administered per 100 people across income groups. Note that the number of individuals who have received at least one booster is lower than the number of boosters administered since some people might have received more than one booster dose. In addition, the decision to administer booster doses, the timing of administration, and the eligible population differed across countries. Notwithstanding all these facts, the graph shows that the administration of booster doses is highly regressive. In low-income countries, the number of administered boosters covers less than 4% of the population, while in high-income countries, the number of administered boosters could cover 66% of the population. This comparison highlights an additional element of inequality in the global allocation of COVID-19 vaccines, as one could easily argue that some of the booster doses could have been used to increase primary vaccination rates in lower-income countries.

The previous figures speak about the inequality in global COVID-19 vaccination rates. To what extent is the unequal distribution of COVID-19 vaccines also inequitable? To answer this question, I take an ex-ante perspective and reflect on what would have been an equitable global allocation of COVID-19 vaccines in the initial phases of the vaccination campaign given the information available at that time concerning COVID-19 epidemiology and anticipated effectiveness of COVID-19 vaccines. In other words, suppose we are back at the beginning of 2021, and we have to decide how to globally allocate the first batches of COVID-19 vaccines. Which factors should we consider to determine an equitable global outcome?

The United Nations defines global vaccine equity as the situation where vaccines are “allocated across countries based on needs and regardless of their economic status” [28]. The crucial issue here is how to characterize “needs” for COVID-19 vaccines. I am going to take for granted that the populations with the largest need are those who are expected to suffer the most from COVID-19 if not vaccinated. However, harm can be measured in multiple ways. Direct harms of COVID-19 include deaths, disabilities, and hospitalizations caused by COVID-19 infections. Thus, countries with the largest need may be those that anticipate the largest health burden if their population is not vaccinated. But COVID-19 causes also indirect harms, including additional deaths and disabilities due to strains in the healthcare system, unemployment, business closures, educational gaps, and the risk of falling into poverty. Therefore, countries with the largest need may be those that anticipate the largest socioeconomic burden if their population is not vaccinated.

At least four additional factors complicate the definition and measurement of “needs” for COVID-19 vaccines. First, the lack of COVID-19 vaccination creates both health and non-health harms. This requires a methodology for weighing the relative importance of qualitatively different harms [29]. For instance, since the beginning of the pandemic, it was clear that mortality and severe health consequences were positively associated with age [30]. On the other hand, the socioeconomic burden of the pandemic fell largely on children and working-age adults from low socioeconomic backgrounds due to disruptions in the education system and labor markets [31–33]. In addition, less resilient economies are expected to suffer more from the pandemic (and less resilient economies typically coincide with younger and poorer populations) [34]. Should the distribution of COVID-19 vaccines aim at reducing the health burden first and foremost, or should it aim at saving livelihoods and reducing the economic burden? The former would justify sending the vaccines first to countries with an older population, while the latter would justify sending the vaccines first to countries that face the largest risk of poverty due to COVID-19. Note also that income reductions tend to have detrimental effects on population health (e.g., individuals forego prevention activities and treatments because of liquidity issues, thereby putting them at higher risk of morbidity and mortality in the future). Thus, there is not only a trade-off between saving lives and saving livelihoods but also between saving lives now and saving lives in the future.

Second, and related to the previous issue, overall harms can be reduced by increasing the resilience of healthcare systems and welfare programs. For example, a country with a large share of older adults but a good healthcare system may face overall lower mortality rates than a country with a smaller share of older adults but a less resilient healthcare system. Similarly, the coverage and generosity of welfare programs (e.g., unemployment subsidies) can substantially reduce the socioeconomic costs of the pandemic, thereby weakening the economic argument in support of vaccination prioritization. This calls for skewing the allocation of vaccines towards low-income countries, as they are less likely to be able to cope with the negative effects of a pandemic.

Third, the optimal allocation of COVID-19 vaccines is complicated by the potential effectiveness of the vaccine in reducing transmission risks. Given the network of relations and contacts among countries and populations, protecting the needs of the most vulnerable may call for vaccinating first less vulnerable, but highly connected populations. For example, debates about the optimal allocation of COVID-19 vaccines within a country were shaped by the trade-off between vaccinating the older people first (high risk, low contacts) or the working-age population first (lower risk, high contacts) [35]. The same argument can be extended at the global level: Should vaccines be allocated to countries that are facing the largest harm (however defined) or to countries that are more likely to transmit the virus to the rest of the world? Even if one believes that poorer countries have less need for COVID-19 vaccines due to their demographics (i.e., low share of older people), distributing vaccines in low-income countries could in principle prevent the surge of new variants and new waves of infections, whose negative impacts will likely be transmitted to wealthier countries.

Finally, countries’ relative “need” for COVID-19 vaccines is not a static concept, but it changes over time depending on the proportion of people already infected or vaccinated (i.e., the proportion of people that are presumed to be protected at least in the short-term), on the characteristics of the dominant virus variant (e.g., its transmissibility and lethality), on the expected future trends of virus transmission channels, and on the capacity of a country to sustain new pandemic waves from both socioeconomic and healthcare perspectives (e.g., the experienced degree of disruption of the healthcare system and the state of public finances after prolonged public economic support).

Determining the optimal and equitable global allocation of COVID-19 vaccines is beyond the scope of the paper. A few contributions have considered this issue and proposed frameworks to guide the distribution of vaccines and to judge its reliance on principles of fairness. A noteworthy proposal is the “fair priority model” that suggested replacing the proportional allocation (by population size) from COVAX with an allocation based on the urgency of needs [36–38]. The fair priority model envisions three phases of vaccine allocation, with the goals, respectively, of reducing premature deaths, reducing serious economic and social deprivation, and reducing community transmission.

Among the factors to consider in determining the equitable global allocation of COVID-19 vaccines, I listed also the potential effectiveness of vaccines in reducing transmission. The importance of the transmission-reducing goal in COVID-19 vaccine allocation is nowadays an open question. Although existing COVID-19 vaccines were found to be partially effective at preventing transmission of the initial virus strains [39], vaccine-associated reductions in transmission of the new variants are considerably lower and rapidly wane over time [40], leading to larger numbers of breakthrough infections. On the other hand, even if not perfectly shielded from infectiousness, vaccinated individuals appear to be less infectious than unvaccinated individuals and present reduced and faster-disappearing infectious viral load [41–43]. All this considered, should differences in transmission risk across countries matter in determining the largest need for COVID-19 vaccines? I argue that a retrospective evaluation of the inequity in global COVID-19 vaccinations should reflect the information available in the initial phases of vaccine distribution when the potential effects of COVID-19 vaccines on reducing transmission were deemed to be positive. Instead, prospective evaluations (i.e., how COVID-19 vaccines or other pandemic vaccines should be distributed in the future) should account for the most recent information on the characteristics of vaccines.

**Fig 3:**
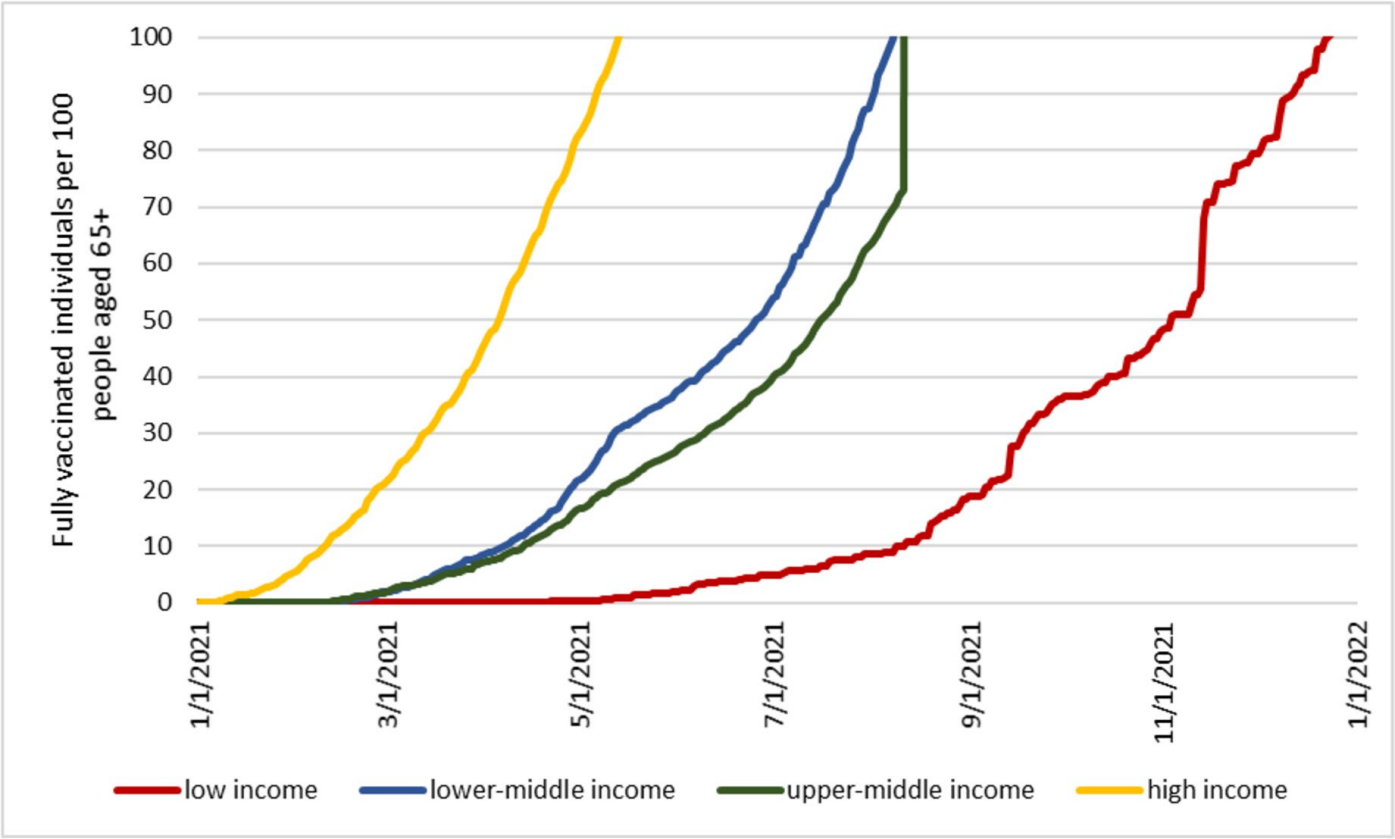
Number of fully COVID-19 vaccinated individuals as a percentage of people aged 65 and over. Source: Numbers of fully vaccinated individuals are from our World in Data [20]. The total population aged 65 and over is from United Nations World Population Prospects 2022 (2021 data) [44]

Although inequality is not necessarily synonymous with inequity, the stark differences in vaccination rates across country income groups, the large health and economic toll suffered by unvaccinated populations, and the current lack of constraints in global vaccine supply all indicate that the unequal distribution of COVID-19 vaccines and vaccination rates has indeed been inequitable and suboptimal. To reinforce this argument, Figure 3 shows the number of fully vaccinated individuals as a percentage of the population aged 65 and over by country income group. The share of older people is an imperfect metric of the population at highest risk of COVID-19 hospitalization and death. Mortality from COVID-19 is correlated not only with age, but also with the presence of comorbidities, pollution levels, and living arrangements, among other factors [45–47]. Due to the nature of their job, frontline healthcare workers and other essential workers were also more likely to get infected and suffer severe health consequences independently of their age [48]. In addition, as previously discussed, COVID-19 mortality and morbidity encompass only a fraction of the possible harms caused by the pandemic.

The figure shows that, by the end of April 2021, high-income countries had distributed enough vaccines to cover the population aged 65+ (i.e., presumably the population at highest risk). In contrast, low-income countries reached that goal only 8 months later, and middle-income countries about 3 months later. Considering the smaller proportion of older people in lower-income countries (3.1% in low-income countries and 6.0% in lower-middle-income countries compared to 18.9% in high-income countries and 11.8% in upper-middle-income countries), the difference in protection is remarkable. Of course, not all vaccine doses were administered preferentially to older people. Some of those initial vaccine doses were given to frontline healthcare workers, individuals with comorbidities, and other individuals who were considered essential or worthy of prioritization. Still, the graph shows that, for many months, the number of fully vaccinated people in lower-income countries was substantially lower than the number of people considered at most risk. Data from COVAX confirm that by August 2021, only 33 million COVID-19 vaccine doses were delivered to low-income countries compared to 1.6 billion in high-income countries [22], thereby suggesting that the slow vaccine uptake in low-income countries in the first half of 2021 was foremost due to supply issues.

## Barriers to vaccine equity

Different factors contribute to global inequity in COVID-19 vaccination rates. Vaccine nationalism, i.e., the stockpiling of vaccines by high-income countries, has been a major issue since the beginning of the COVID-19 vaccination rollout [49]. Facing a limited global supply of COVID-19 vaccines, countries with manufacturing capacity and other high-income countries tended to prioritize their populations rather than slowing the spread of COVID-19 elsewhere [50]. Despite pledges from developed countries to donate vaccines to low- and middle-income countries, only a fraction of the promised doses has been delivered due to export bans on vaccines and vaccine ingredients [51]. The lack of a globally coordinated approach to vaccine allocation implied that vaccination in lower-income countries started months later and has proceeded at a slower pace than vaccination in higher-income countries. The emergence of new variants and the waning of vaccination-acquired immunity have strengthened vaccine nationalism, with vaccines directed to higher-income countries for booster doses, thereby further delaying their availability in lower-income countries.

A deeper problem underlying vaccine inequity is the unequal distribution of vaccine manufacturing capacity. With a few exceptions (e.g., China, Cuba, India), low- and middle-income countries did not have manufacturing capacities in place at the beginning of the pandemic [52]. Although there have been some investments in vaccine manufacturing in lower-income countries (e.g., the WHO’s vaccine technology transfer hub at Afrigen Biologics and Vaccines in Cape Town that allowed the replication of mRNA vaccines in 2022 [53]), manufacturers’ reluctancy to share the knowhow has constrained the overall production capacity as well as hampered global access to COVID-19 vaccines [54]. As a consequence, poor countries with no manufacturing capacity have to rely on international vaccine supplies, encompassing both vaccine donations and market purchases.

The affordability of vaccines is another constraint to increasing vaccination in lower-income countries. The cost of COVID-19 vaccines varies widely across countries, depending on the product characteristics and bilateral agreements between pharmaceutical companies and countries. According to WHO’s estimates, the price per vaccine dose ranges from US$2 to US$40 [55], while the cost of delivering COVID-19 vaccines in low- and middle-income countries is around US$3.70 per vaccinated person [56]. Delivery costs include planning and coordination, outreach initiatives, cold chain equipment, vaccine transport and training, and accounting for possible wastage. Considering that health expenditures per capita in low-income countries are around US$35 [57], COVID-19 vaccines represent a significant financial burden. The WHO estimates that vaccinating an additional 40% of the population in low-income countries would require on average between 10% and 25% of their annual healthcare budget [58].

Differences in healthcare systems capacity, including health workforce, vaccine supply chains, and infrastructures, contributed to global vaccine inequity. For instance, COVID-19 vaccines based on mRNA technologies require ultra-cold chains; maintaining a cold chain is problematic for hard-to-reach rural areas with irregular electricity supply [59]. It is worth noting that mRNA technologies contributed to the accelerated development and manufacturing of COVID-19 vaccines, and, in their absence, the pandemic would probably have had more devastating impacts on the global population and exacerbated existing health inequities [18]. However, the ultra-cold chain requirements are clearly an obstacle to global vaccine equity. Another barrier to vaccine equity is the potential shortage of equipment (e.g., syringes), which constantly threatens vaccination efforts, especially in lower-income countries [60, 61]. Support from local health workers is also pivotal for delivering vaccines in countries with less resilient healthcare systems [62]. Lack of resources for continuous training, compensation, and technical support of the health workforce can thus undermine vaccination efforts.

Although supply constraints were the main barriers to vaccine equity in the first months of the COVID-19 vaccination rollout, vaccine hesitancy contributes to the current inertia in vaccination rates. Vaccine hesitancy is fueled by misperceptions of COVID-19 vaccine safety and effectiveness, misperceptions about the protection afforded by previous exposure, and mistrust in the institutions responsible for vaccine communication and delivery [63–65]. A recent World Bank report found that about 20% of adults in developing countries are hesitant about getting COVID-19 vaccination [66]. The reported COVID-19 vaccine hesitancy is also higher than hesitancy towards other vaccines probably because of the novelty of the disease and the associated vaccines.

## Costs of vaccine inequity

Costs of vaccine inequity fall both on the countries with low COVID-19 vaccination rates and on the rest of the world that is already experiencing high vaccination rates. A slow and delayed vaccine rollout in lower-income countries has left them exposed to new surges of the virus and a slower economic recovery from the pandemic. In addition, low vaccination rates in poorer countries may produce global health and economic negative externalities.

Although high-income countries have larger shares of older people (i.e., the individuals considered more vulnerable to COVID-19), low-income countries have less resilient healthcare systems, which make them more vulnerable to disruptions in access and delivery of healthcare, and, as a result, to increasing morbidity and mortality burdens. For example, COVID-19 has caused substantial negative impacts on the management of endemic diseases (e.g., HIV, malaria, neglected tropical diseases), and on childhood immunization programs [67–71]. The future health burdens of these disruptions (e.g., the number of healthy life years lost due to COVID-19) are yet to be determined. Furthermore, the lack of access to good-quality healthcare often translates into larger age-specific COVID-19 infection fatality rates in developing countries compared to developed ones [72].

Global fair distribution of vaccines could have reduced the health burden in lower-income countries, and it may prevent future burdens. For instance, it was estimated that COVID-19 vaccine hoarding might have cost more than 1 million lives in 2021 [73]. If countries had started to share vaccines at the beginning of the vaccination rollout, and vaccines had been distributed proportionally to the size of the adult population, fewer infections would have occurred in low- and middle-income countries and, as a result, fewer COVID-19 deaths. These outcomes are counterbalanced by an increase in infections in high-income countries (although not necessarily an increase in deaths if high-risk populations are vaccinated first) and, potentially, an increase in economic costs in high-income countries if nonpharmaceutical interventions are enforced for a longer period.

**Fig. 4:**
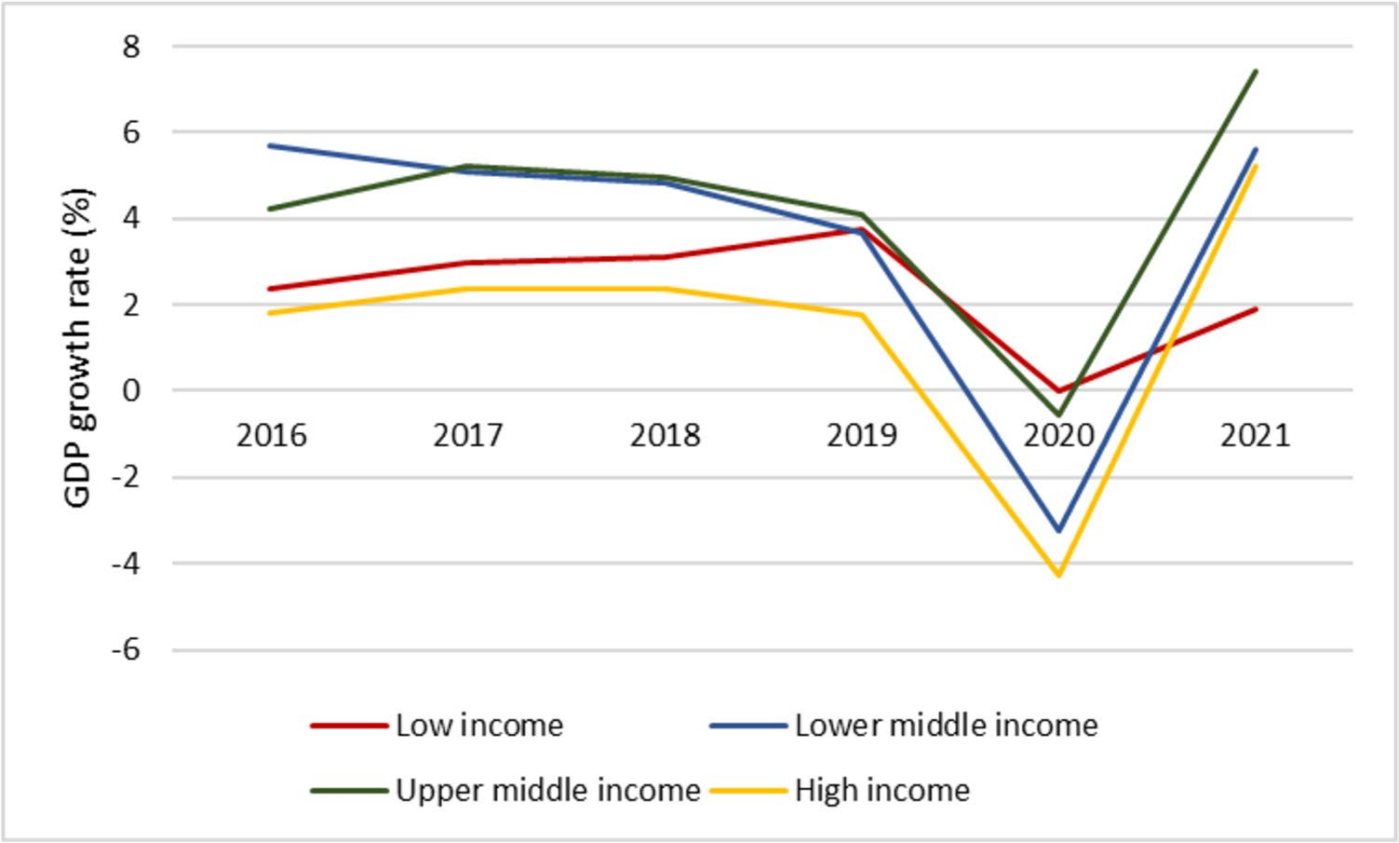
Gross domestic product annual growth rate (%). Source: World Bank Indicators [76]

Low vaccination rates in lower-income countries have also been responsible for a slower recovery. In countries with early and fast vaccine rollout, COVID-19 vaccination allowed a progressive reopening of the economy, with associated increases in consumer spending, employment rates, and overall gross domestic product (GDP) [74, 75]. In contrast, countries with low vaccination rates endured relatively longer periods with economic lockdowns and containment measures in place [25]. While advanced economies suffered larger economic losses at the beginning of the pandemic, lower-income countries bear the brunt of the COVID-19 economic burden in 2021. Figure 4 depicts the annual GDP growth rate across country income groups. Low-income countries enjoyed a slower GDP growth compared to the rest of the world, and the GDP growth rate in 2021 was still substantially lower than pre-pandemic growth rates. Had COVID-19 vaccines been distributed more fairly, poor countries would have experienced a faster recovery.

Besides health and economic costs, the COVID-19 pandemic has caused human capital degradation throughout the world, especially in lower-income countries [15, 77]. Almost 1.6 billion students around the world were affected by school closures because of the COVID-19 pandemic [78]. Even short disruptions in schooling can have significant negative effects on learning, and students in disadvantaged families and communities are more vulnerable to these disruptions [79]. Learning losses translate also into long-term productivity losses, thereby threatening children’s future livelihoods and countries’ future macroeconomic performance. Poorer countries reported the longest average duration of school closure, thereby widening the learning gap between rich and poor countries [80]. Low vaccination rates increase the risk of school closures and make it harder to overcome the learning losses because of limited financial resources triggered by the slow economic recovery.

Furthermore, increasing vaccination rates in poorer countries produces benefits for the entire world. First of all, higher-income countries have an economic incentive to provide equitable access to COVID-19 vaccines [81]. In an economically interconnected world, preventing waves of infections in poorer countries reduces the risk of global supply chain disruptions, which would negatively impact the domestic economy of high-income countries. In particular, potential economic lockdowns in lower-income countries triggered by a lack of vaccination hurt higher-income countries by causing a shortage of intermediate inputs, higher import prices, and weak demand for their exports [82].

In addition, it has been suggested that global COVID-19 vaccine inequity translates into an increased risk of the spread of new immune-evasive variants [83, 84]. The argument is that new strains are more likely to spread in populations with low vaccination rates. These new strains will then be exported to other countries and cause new waves of infections worldwide since the vaccine is expected to offer only partial protection against new variants. Given the current understanding of the epidemiology of COVID-19 and the characteristics of COVID-19 vaccines, the strength of this argument is debatable. On the one hand, as pointed out earlier, existing COVID-19 vaccines are very effective at preventing severe disease and death, but not very effective at preventing infection and transmission, especially with the new variants [39, 40]. Effectiveness also wanes rapidly [85]. As a result, existing COVID-19 vaccines provide mostly direct benefits, while the potential benefits in curbing the spread of new strains seem to be minimal. In that case, new strains can emerge and rapidly spread even in populations with high vaccination rates, and reducing global COVID-19 vaccine inequity produces no positive health externality. On the other hand, there is some evidence that infectious viral load is lower among vaccinated than unvaccinated individuals [42] and that viral load clears faster among vaccinated people [43]. Although the transmission process of COVID-19 is complex, viral load is recognized as a strong determinant of transmission risk [86], thereby suggesting that new variants are more likely to spread among unvaccinated individuals. This viral load channel would then support the assertion that global COVID-19 vaccine inequity leads to the development of new more dangerous variants. However, to my knowledge, there is yet no evidence to support this conclusion, and the potential global health costs of COVID-19 vaccine inequity are an open question.

## Conclusions

The paper has presented evidence of global COVID-19 vaccine inequity and discussed the main factors contributing to this inequity and its health, social, and economic consequences. In particular, the paper highlighted how vaccine inequity harms both the countries experiencing low vaccination rates and the countries with high coverage. These indirect effects are the result of social and economic connections across countries. Thus, it is in the self-interest of high-income countries to improve global access to COVID-19 vaccines. Yet, vaccine inequity is still an issue.

Several lessons can be drawn from the analysis of the paper. These lessons can be useful for the next stages of the COVID-19 pandemic and for preparing for the (likely) next one.

First of all, COVID-19 vaccines were developed and manufactured at accelerated speed. This was in part the result of the massive amount of public financial resources dedicated to the project. Taking the USA as an example, estimates suggest that the government invested $18 billion through Operation Warp Speed for securing COVID-19 vaccine doses and for financing development and manufacturing capacity [87]. Public financing of vaccine R&D and manufacturing capacity has the purpose of partially absorbing the risk of vaccine development, thereby enhancing pharmaceutical companies’ incentives to invest in vaccines. Indeed, vaccines constitute a high-risk investment, since their development carries a substantial risk of failure, and their profitability is linked to an uncertain demand, often from lower-income countries with low ability to pay [88, 89]. In addition, vaccine development, testing, manufacturing, and distribution typically require long and highly variable periods of time, during which the destructive potential of a pandemic can accelerate. In preparation for the next pandemic, it is thus essential to pour public funding into accelerating the vaccine development process, e.g., by investing in pre-pandemic times in the development of prototype vaccines for pathogens with pandemic potential and by enhancing global vaccine manufacturing capacity (independently of its location) [90, 91]. These investments reduce the risk of global vaccine supply constraints when the next pandemic strikes. In particular, by easing the global supply constraints, there will be less international competition for limited doses and, as a result, more vaccines for lower-income countries.

Although funding the accelerated global production of vaccines against the next pandemic may reduce global vaccine inequity by itself, the development of local manufacturing capacity will further enhance global vaccine equity. Indeed, less developed countries need to have access to local manufacturing capacity to avoid being dependent on donations and excessive production of higher-income countries. The development of local manufacturing capacity requires the waving of intellectual property rights and technology transfer agreements, despite potential resistance from pharmaceutical companies. The WHO vaccine hub in South Africa is an example of mRNA technology transfer [53], but more initiatives are imperative. Underinvestment in local manufacturing capacity perpetuates the inequity in access to vaccines and other medical products and, as a result, has the potential to trigger global health and socioeconomic costs. As stated by the Managing Director of the International Monetary Fund, Ms. Kristalina Georgieva, “support for Africa’s vaccine production is good for the world” [92].

Moreover, investments to strengthen healthcare systems are necessary to allow effective and timely delivery of vaccines. Logistical barriers, such as maintaining ultra-cold chains and reaching remote areas, have hampered the process of vaccine distribution in lower-income countries. Strong health systems require investments in new and improved equipment, infrastructure, monitoring and surveillance systems, data collection, and training of healthcare workers. The benefits of these investments are clearly huge and go beyond the realm of pandemic preparedness.

Strategies to overcome vaccine hesitancy are also welcome. There are different reasons underlying vaccine hesitancy, from mistrust in authorities or science to misunderstanding the available information. Here the role of local communities and trustworthy figures is fundamental. Investments in outreach initiatives led by local health workers should be a key element of any pandemic preparedness strategy.

Since vaccination is a public good (and infectious diseases are a public bad), the cooperation of all countries is necessary to guarantee global vaccine equity and a swift response to future pandemic threats. Nationalistic incentives can be overcome only through a binding international agreement [93, 94], where countries commit to share knowledge and technologies, strengthen the healthcare systems, and contribute to the research and development of vaccines and other products against pathogens with pandemic potential. Funding for such an initiative should be managed by a supranational authority that receives contributions from multiple countries and allocates pandemic preparation spending across different projects, along the lines of the recently established WHO- and World Bank-supported Financial Intermediary Fund for pandemic prevention, preparedness, and response [95].

Future pandemics may be more devastating than the COVID-19 one, especially for lower-income countries. Effective pandemic preparedness requires, among other things, strategies to overcome existing barriers to global vaccine equity. Failing to do that could be tragically shortsighted.

## Data Availability

The data used in this study are derived from public domain resources. References to those sources are available within the article.
